# Brain-Derived Neurotrophic Factor (BDNF) Is Associated with Platelet Activity and Bleeding Tendency in Patients with Gaucher Disease

**DOI:** 10.3390/ijms232213982

**Published:** 2022-11-12

**Authors:** David Azoulay, Mira Naamad, Dafna Frydman, Ellen Broide, Ari Zimran, Galia Stemer, Shoshana Revel-Vilk

**Affiliations:** 1 Hematology Unit and Laboratories, Galilee Medical Center, Nahariya 22100, Israel; 2 Azrieli Faculty of Medicine, Bar-Ilan University, Safed 1311502, Israel; 3 Flow Cytometry Unit, Shaare Zedek Medical Center, Jerusalem 9103102, Israel; 4 Gaucher Unit, Shaare Zedek Medical Center, Jerusalem 9103102, Israel; 5 Faculty of Medicine, Hebrew University of Jerusalem, Jerusalem 9112002, Israel; 6 Pediatric Hematology/Oncology Unit, Shaare Zedek Medical Center, Jerusalem 9103102, Israel

**Keywords:** Gaucher disease (GD), brain-derived neurotrophic factor (BDNF), alpha degranulation defect, platelets

## Abstract

Bleeding tendency, a prominent feature of patients with Gaucher disease (GD), is associated with abnormal platelet function. Brain-derived neurotrophic factor (BDNF) is a protein with neuroprotective potential stored in alpha granules of circulating platelets. Here we studied BDNF levels in 50 patients with type I GD (GD1) and their correlation with platelet activity and bleeding tendency. Flow cytometry was used to test unstimulated and stimulated measurement of platelet surface-activated expression of αIIbβ3 integrin, P-selectin and lysosomal-associated membrane protein (LAMP3/CD63). Serum and plasma BDNF levels were quantified using ELISA. The bleeding history was recorded by a bleeding questionnaire. Serum BDNF levels were positively correlated with platelet count and moderately correlated with unstimulated and stimulated platelet P-selectin expression. Patients with more than one bleeding manifestation were shown to have lower serum BDNF levels, albeit similar platelet count. Plasma BDNF levels were significantly elevated in splenectomized patients and showed a moderate positive correlation with stimulated platelet CD63 expression. These observations demonstrate the first association between BDNF levels in the peripheral blood with platelet dysfunction and increased bleeding manifestation. The role of measuring serum BDNF for assessing platelet alpha degranulation defects and bleeding risk in patients with GD and the general population needs further study.

## 1. Introduction

Gaucher disease (GD), the most common lysosomal storage disease, is characterized by the accumulation of glucosylceramide-laden macrophages in various organs, most notably of the reticuloendothelial system leading to hepatosplenomegaly, anemia, thrombocytopenia and bone infarction [1]. It is an autosomal recessive disease caused by functional deficiency of the lysosomal enzyme β-glucocerebrosidase, resulting from variants in the *GBA1* gene. Worldwide, the incidence of GD is approximately 1 in 50,000 to 100,000 live births; however, due to a high carrier frequency of 6% in Ashkenazi Jews, studies indicate a homozygote frequency of ~1:850 in the Ashkenazi Jewish population. Gaucher disease is typically categorized into three main types; type 1, a non-neuronopathic most prevalent variant among Caucasian patients, type 2, an acute neuronopathic variant occurring in very young children and type 3, a subacute neuronopathic variant that tends to manifest neurologically in childhood or adolescence [1]. The diagnosis of GD is based on low enzyme β-glucocerebrosidase levels, high glucosylsphingosine (lyso-Gb1) levels and molecular analysis of the *GBA1* gene [2]. The currently available treatment for GD, including enzyme replacement therapy and substrate reduction therapy, are effective in reducing disease burden [1].

Bleeding tendency is a prominent feature of patients with GD, commonly attributed to thrombocytopenia and coagulation abnormalities [3,4]. However, evidence of patients having a clinically significant bleeding tendency, not proportional to their platelet counts or coagulation abnormalities, led to the diagnosis of platelet dysfunction in patients with GD [5,6,7].

Platelet activity test by whole blood flow cytometry (FC) is a powerful method to study platelet function. In this method, platelets are directly analyzed in their physiological microenvironment, which consists of red blood cells and white blood cells, avoiding redundant separation and washing steps that may affect platelet activation [8]. More importantly, in the FC method, platelets of patients with profound thrombocytopenia can also be accurately analyzed, a major advantage when treated and untreated adults and children with GD are tested [9]. Recently, Revel-Vilk and colleagues established a method for evaluating FC-based platelet function in a large cohort of patients with GD. This method showed that the platelet dysfunction in GD was related to reduced P-selectin expression, i.e., alpha degranulation defect [10].

Brain-derived neurotrophic factor (BDNF) is a member of the neuronal growth factors family, which includes nerve growth factor (NGF), and neurotrophin (NT) 3/4 and 5 [11]. Platelets contain BDNF in the cytoplasm and alpha granules, and are considered the primary storage of BDNF [12]. Several leukocyte subsets, such as T and B lymphocytes and monocytes, have also been shown to produce and secrete BDNF when activated by different stimuli [13,14,15]. In humans, high levels of BDNF protein can be detected in the serum and the plasma [16]. The serum BDNF level is believed to represent the total BDNF content discharged from platelets, and it is detected at levels about 10-fold more than in the plasma [17,18].

As platelet alpha granules are considered to be the main compartment for BDNF storage and mobilization, BDNF seems to be a good candidate biomarker to investigate platelet dysfunction in GD. However, the relationship between platelet activation and blood BDNF levels is poorly studied in humans [19]. Two studies that investigated plasma BDNF in patients with GD indicated reduced levels in patients with type I GD (GD1) [20], and restoration of plasma BDNF levels in patients treated with enzyme replacement therapy (ERT) [21]. However, these studies did not investigate serum BDNF level. Furthermore, the interaction of BDNF and bleeding phenomena, platelet count and/or function in the general population and patients with GD was not studied. Thus, in this study, we were interested in testing the hypothesis that serum and plasma BDNF levels are associated with platelet function and bleeding tendency in patients with GD1.

## 2. Results

Fifty patients with GD1 (25 males) were included in this study. Most patients were treated with GD-specific therapy, i.e., ERT or substrate reduction therapy (SRT), and only three patients were splenectomized (Table 1). Splenectomies were done prior to availability of GD-specific therapy.

### 2.1. Serum BDNF Levels Positively Correlate with Platelet Count and Unstimulated and Stimulated Platelet P-Selectin Expression

Serum BDNF levels were normally distributed in the patients (*p*(W) = 0.17) with a mean ± SD of 10.72 ± 4.17 ng/mL, and a range of 3.23–18 ng/mL. A weak negative correlation was found between serum BDNF level and age (r = −0.27, *p* = 0.055). Serum BDNF levels were not associated with disease severity by genotype, use of GD-specific therapy, history of splenectomy, and glucosylsphingosine (lyso-Gb1) levels.

**Figure 1:**
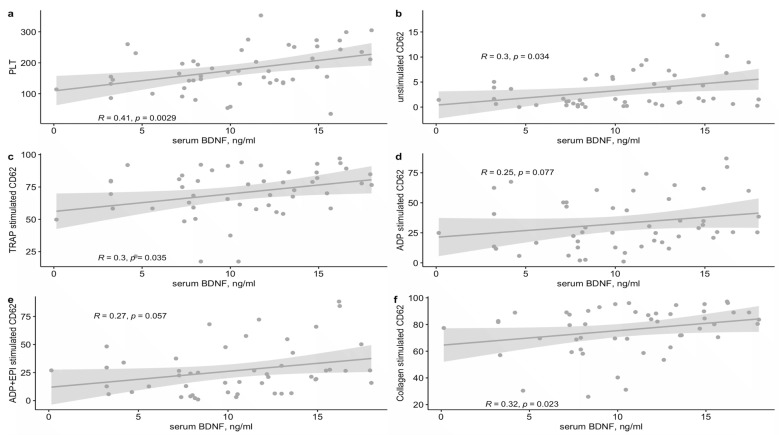
Correlation of serum BDNF levels with (**a**) platelet count, (**b**) unstimulated platelet P-selectin (CD62) expression and (**c**–**f**) stimulated platelet P-selectin (CD62) expression. Scatter plots are shown, with a regression line and a 95% confidence interval. BDNF, brain-derived neurotrophic factor; PLT, platelet count, ×10^9^/mL; TRAP, the protease-activated receptor (PAR)-1 agonist thrombin receptor activator peptide; ADP, adenosine diphosphate; EPI, epinephrine.

A positive correlation was found between serum BDNF levels and platelet count, and a moderate correlation was found with unstimulated P-selectin expression, protease-activated receptor (PAR)-1 agonist thrombin receptor activator peptide (TRAP) stimulated platelet P-selectin expression, and collagen stimulated platelet P-selectin expression (Figure 1). Serum BDNF levels were not correlated with unstimulated and stimulated platelet alphaIIbetaIII integrin or CD63 expression. Patients with high serum BDNF (i.e., >14 ng/mL) levels showed significantly higher P-selectin expression to stimulation by TRAP and collagen and higher CD63 expression to stimulation by TRAP as compared to patients with low serum BDNF levels (i.e., <7.8 ng/mL) (Table 2). Expression of alphaIIbetaIII integrin was not associated with high vs. low serum BDNF levels.

### 2.2. Plasma BDNF Levels Are Increased in Splenectomized Patients and Positively Correlated with Stimulated Platelet CD63 Expression in Patients with an Intact Spleen

**Figure 2:**
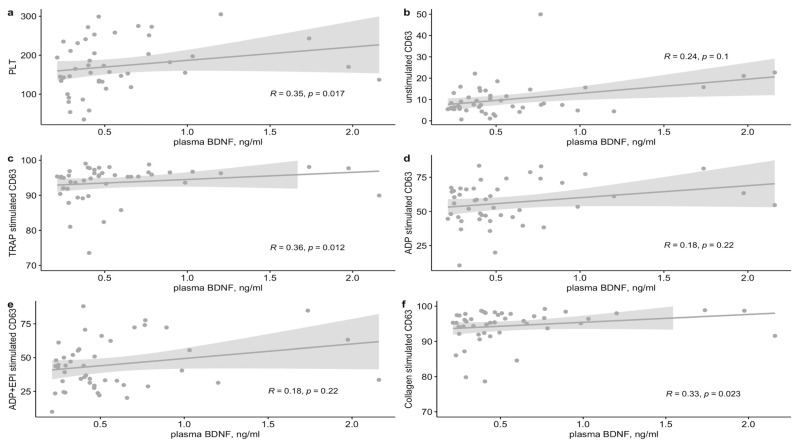
Correlation of plasma BDNF levels with (**a**) platelet count, (**b**) unstimulated platelet CD63 expression and (**c**–**f**) stimulated platelet CD63 expression. Scatter plots are shown, with a regression line and a 95% confidence interval. BDNF, brain-derived neurotrophic factor; PLT, platelet count, ×109/mL; TRAP, the protease-activated receptor (PAR)-1 agonist thrombin receptor activator peptide; ADP, Adenosine diphosphate; EPI, epinephrine.

Plasma BDNF levels were not normally distributed (*p*(W) < 0.0001) with a median (IQR) of 0.45 (0.3–0.61) ng/mL. As splenectomized patients had significantly higher plasma BDNF levels, median (IQR) 1.19 (0.9–1.58), compared to those with intact spleens, median 0.44 (0.29–0.65) (*p* = 0.035), further analysis was done only on patients with an intact spleen. Plasma BDNF levels were not associated with age, disease severity, GD-specific therapy or glucosylsphingosine (lyso-Gb1) levels. A moderate positive correlation was found between plasma BDNF levels and platelet count, TRAP-stimulated platelet CD63 expression and collagen-stimulated platelet CD63 expression (Figure 2). No correlation was found with unstimulated or stimulated αIIbβ3 integrin and P-selectin expression. Patients with high plasma BDNF (i.e., >0.45 ng/mL) levels showed significantly higher CD63 expression to stimulation by TRAP and collagen as compared to patients with lower levels (Table 2). Expression of alphaIIbetaIII integrin and P-selectin were not associated with high vs. low plasma BDNF levels.

### 2.3. Patients with More Than One Bleeding Manifestation Show Lower Serum BDNF Levels, Albeit Similar Platelet Counts

Bleeding questionnaires were completed by 36 patients (Table 1). Patients with more than one bleeding symptom were found to have significantly lower serum BDNF levels compared to patients with one or no bleeding symptoms (Table 3). Notably, more than one bleeding symptom was not associated with platelet counts, thrombocytopenia, disease severity by genotype, therapy or plasma BDNF levels.

## 3. Discussion

In this study, we intended to examine the association between BDNF levels, platelet activity and bleeding tendency in patients with GD1. We found that serum BDNF levels were associated with platelet P-selectin expression and a bleeding tendency. Plasma BDNF levels were associated with splenectomy and platelet CD63 expression.

Serum BDNF, detected at relatively high levels, represents the total BDNF content extracted from the platelets in the process of serum isolation [11,12]. The positive correlation with P-selectin is not surprising, as both BDNF and P-selectin are stored in platelet alpha granules. The platelet alpha degranulation defect in GD [10], leading to lower P-selectin expression, may have led to the lower discharge of BDNF from the platelets to the serum. Alternatively, it could be speculated that patients with high levels of BDNF have more alpha granules per platelet and/or more content of BDNF within their granules, which better facilitate their platelets’ function. However, this postulation and the potential role of serum BDNF as an indicator of platelet alpha degranulation defect need to be further studied.

As far as we know, our study is the first to report a link between serum BDNF levels and bleeding tendency. In GD, it is consistent with our previous findings that a platelet degranulation defect was related to the bleeding tendency [10]. Whether this association will also be found in a non-GD cohort still needs to be studied. The effect of BDNF on the coagulation system, mainly on clot stability and the balance between fibrinogenesis and fibrinolysis, was recently indicated [22]. Therefore, it is possible that the low serum BDNF is not only a marker of platelet degranulation defect, but contributes to the bleeding tendency by affecting the coagulation system. Practically, we think that the measurement of serum BDNF levels in patients with GD could be developed into an additional and complementary laboratory tool for the evaluation of bleeding risk. However, this direction should be addressed by further studies and verified by prospective data.

In contrast to BDNF levels in the serum, BDNF levels in the plasma were associated with platelet CD63 expression. We also observed the increase of plasma BDNF, but not the serum BDNF, in splenectomized patients. CD63 expression was shown to increase in platelets under an inflammatory state [23,24,25] and was strongly associated with the secretion of inflammatory granules from immune cells [26]. Platelet CD63 expression has also been considered a sign of inflammation in GD [10]. As the plasma we isolated was platelet-poor plasma, we assume that the BDNF content in the plasma represents free soluble BDNF levels from other resources, such as activated immune cells [15]. While the mechanism behind the elevated plasma BDNF levels in splenectomized patients still needs to be explored, it may be speculated that it is related to the increased inflammatory processes and immune cell activation in these patients. Alternatively, plasma BDNF may represent BDNF attached to the surface of platelets following their activation and, therefore, represent activated platelets that are inefficiently cleared from the circulation of splenectomized patients.

The plasma BDNF levels in patients with GD were previously studied in smaller cohorts. In 14 treated patients (at least six months on ERT), the plasma BDNF levels of 0.39 ng/mL IQR (0.25), were similar to our study. Levels were significantly lower when off ERT (at least four months), at 0.14 ng/mL (IQR 0.09 ng/mL) [20]. This may be explained by the increase in platelet count with ERT. As untreated patients in our cohort are those with the milder phenotype and their mean platelet count was similar to treated patients, we could not find this phenomenon. In 10 patients with GD1, plasma BDNF levels were lower, median (range) 0.128 (0.048–0.17) ng/mL; however, treatment status and splenectomy rate were not reported [21]. A moderate negative correlation was found between plasma BDNF and interleukin-4 (IL-4) and tumoral necrosis factor-alpha (TNF), supporting the inflammatory process in GD. Both studies did not refer to serum BDNF levels and the effect platelet count and function may have on BDNF levels.

Lastly, the association of GD with Parkinson Disease (PD) is one of the most important areas of investigation in GD [27]. BDNF, as a neurotrophic factor, contributes to neuronal cells’ survival, maintenance, and repair [28,29]. A meta-analysis of serum BDNF levels in patients with PD shows reduced BDNF compared to controls in most studies [30]. Indications for platelet function defects, such as increased platelet volume and reduced glutamate uptake, were previously shown in PD [31]. Our findings of platelet dysfunction via P-selectin expression and low serum BDNF could be a possible link between platelet activity defects and reduced neuroprotective potential, which may lead to an increased risk of PD or neurogenic manifestation in patients with GD. A new study that we currently designed may hopefully expose this possible link.

Our study has several weaknesses, one of which is that it does not include a control group. As the levels of BDNF in the serum and the plasma is known to be altered by different physiological and disease conditions, we think that the right control group should have the same background to the investigated group. We compared the BDNF levels in patients with GD to the levels in a healthy control group and found no differences [32]. We believe that extending our observations to non-GD-patients with bleeding risk and platelet dysfunction, will be important to investigate by future studies.

## 4. Materials and Methods

### 4.1. Study Population

In this pilot study, data from 50 consecutive patients with GD1 followed at the Gaucher unit, Shaare Zedek Medical Center, between May 2020 and February 2021, with an FC platelet function test done, were collected for this study. Demographics, clinical data, platelet count and treatment status were extracted from clinic charts. Disease severity was defined by genotypes (mild: N370S (c.1226A > G) homozygous and N370S/R496H (c.1604G) compound heterozygous; severe: all other genotypes). At the time of annual/bi-annual patients’ visits, clinical bleeding history was taken, and FC platelet function was done as part of routine practice to assess for bleeding risk. Clinical bleeding history was assessed using a Hebrew-translated self-administered questionnaire (modified from the International Society on Thrombosis and Haemostasis (ISTH)-Bleeding assessment tool (BAT) [33]. The platelet function was analyzed using an established FC assay previously described [10]. In brief, platelet surface activated αIIbβ3 integrin (PAC1), P-selectin (CD62P) and lysosomal-associated membrane protein (LAMP3/CD63) were measured by whole blood flow cytometry at baseline (unstimulated) and after stimulation with agonists (stimulated). Platelet agonists used included adenosine 5′ diphosphate (ADP) (moLab GmbH, Unna, Germany), the protease-activated receptor (PAR)-1 agonist thrombin receptor activator peptide (TRAP-6 (BACHEM, Dubendorf, Switzerland), the glycoprotein VI (GPVI) agonist collagen-related peptide (cross-linked collagen-related peptide (CRP-XL, Cambcol Laboratories, Ely, UK) and epinephrine (moLab GmbH, Unna, Germany). Quantification of glucosylsphingosine (lyso-Gb1) levels on dry blood spot samples were performed at Centogene™ (Rostock, Germany) according to the previously described methods [34]. Residual serum and platelet-poor plasma samples from patients consenting to the biobank project were immediately stored at −80 °C until their analysis. For serum samples, blood tubes without anticoagulants were incubated at room temperature for 30 min until clotting, followed by centrifugation at 3000 rpm (1942 g) for 10 min. The serum was collected from the upper layer into new 1.5 mL tubes and stored at −80 °C. For plasma samples, blood tubes with anticoagulants were centrifuged at 3000 rpm (1942 g) for 10 min at room temperature. The platelet-poor plasma was collected from the upper layer into new 1.5 mL tubes and stored at −80 °C.

### 4.2. Detection of BDNF Protein Levels in Patient Serum and Platelet-Poor Plasma

Serum and platelet-poor plasma samples that were isolated from the patients on the same day of their FC platelet function test and stored at −80 °C were thawed and diluted in phosphate-buffered saline with 1% bovine serum albumin. The BDNF level in the diluted serum (1:50) and plasma (1:5) were quantified using the DuoSet ELISA Development System kit (R&D System DY278, Minneapolis, MN, USA) according to the manufacturer’s instructions. Serum and plasma BDNF concentrations as Nanogram per milliliter (ng/mL) were calculated using an 8-point standard curve and multiplied by the dilution factor to achieve the fixed concentration in the serum and plasma.

### 4.3. Data Analysis

Normally distributed continuous variables were presented as mean, standard deviations and range. Non-normal distributed continuous variables were presented as median, interquartile range (IQR) and range. Normal distribution was tested using the Shapiro–Wilk test. Nominal variables were presented as ratios or percentages from the total. The ANOVA T-test and the Mann–Whitney U test were used to compare the results of two independent parametric and non-parametric variables, respectively. Pearson and Spearman’s tests were used for correlation analysis between parametric and non-parametric continuous variables, respectively. High and low serum BDNF levels were defined according to the normal distribution using the >75th percentile and <25th percentile cutoff points, respectively. For plasma BDNF, according to the non-normal distribution, the median cutoff point was selected to define high and low. All statistical analyses were performed using JMP (SAS Inc., version 10, Cary, NC, USA) statistical software. A *p* value < 0.05 was considered significant.

## 5. Conclusions

Our findings support the concept that patients with GD have a platelet alpha degranulation defect that can lead to reduced platelet p-selectin expression and low serum BDNF levels. Inflammation, described in patients with GD and post-splenectomy, may be marked by increased plasma BDNF levels. Whether BDNF levels can be measured to assess platelet activity and bleeding tendency will need to be further studied in patients with GD and the general population.

## Figures and Tables

**Table 1:** Clinical and laboratory characteristics of study cohort.

**Study cohort**	50
**Age, mean ± SD (range), years**	42.7 ± 17.93 (8.94–86.55)
**Mild genotype, n**	24
**Receiving GD-specific therapy, n**	40
**Splenectomized, n**	3
**Platelet count (×10^3^/µL), mean ± SD (range)**	178 ± 70 (35–353)
**Thrombocytopenia (<50/<100/<150 × 10^3^/µL), n**	1/5/14
**LysoGB1 (ng/mL), mean ± SD (range)**	122.71 ± 143.15 (10–787)
**Serum BDNF (ng/mL), mean ± SD (range)**	10.72 ± 4.17 (3.23–18)
**Plasma BDNF (ng/mL), mean ± SD (range)**	0.61 ± 0.47 (0.21–2.16)
**Bleeding symptoms for those who completed the bleeding questionnaire**	
**0**	17/36
**1**	11/36
**2**	4/36
**3**	3/36
**≥4**	1/36

GD, Gaucher disease; BDNF, brain-derived neurotrophic factor.

**Table 2:** Platelet surface activated alphaIIbetaIII integrin, P-selectin and lysosomal-associated membrane protein (LAMP) expression in patients with high and low serum and plasma BDNF levels.

	Low Serum BDNF	High Serum BDNF	*p*–Value	Low Plasma BDNF	High Plasma BDNF	*p*–Value
***αIIbβ3 integrin (PAC1)***						
Unstimulated	5.7 [1.2–8.5]	5.8 [4.5–9.6]	0.514	4.9 [2.3–8.1]	5.8 [3–8.8]	0.624
ADP (0.5 μM)	75.1 [68.6–85.6]	82.6 [55.6–89.6]	0.828	69.6 [46.7–86.2]	74.1 [51.9–80.9]	0.798
ADP (20 μM)	95 [75.6–97.3]	94.4 [91.4–97.3]	0.744	93.6 [74.3–97.7]	93.6 [77.7–96.9]	0.654
TRAP (1.5 μM)	95.2 [83–96.5]	97.9 [70.6–98.8]	0.355	84.7 [79.3–97.4]	88.3 [76.4–96.5]	0.873
TRAP (20 μM)	97.9 [91.7–98.8]	99.2 [94.3–99.6]	0.128	98.3 [91.7–99.2]	97.7 [90.8–99.2]	0.594
EPI–ADP (0.1–0.25 μM)	88.5 [58.4–92.9]	94.2 [85.7–97.6]	0.073	85.1 [75–94.9]	87.3 [70–93.1]	0.798
CRP–XL (0.25 μg/mL)	97.2 [93.8–98.4]	98.9 [92.5–99.4]	0.328	98.4 [93.9–99.4]	98.4 [96.2–99.1]	0.749
***P–selectin (CD62)***						
Unstimulated	1.2 [0.6–1.7]	3 [1.5–9.3]	**0.036**	0.9 [0.4–3.8]	2.8 [1.1–6.2]	0.068
ADP (0.5 μM)	11.6 [3.8–23]	15.9 [9.2–39.2]	0.301	5.3 [2.8–18.2]	9 [7.1–22.2]	0.268
ADP (20 μM)	24.9 [13.6–50.3]	33.2 [25.6–60.5]	0.192	25.1 [16.7–50.3]	30 [14.8–44.4]	0.609
TRAP (1.5 μM)	46.9 [28.2–58.2]	70.7 [55.3–80.8]	0.073	46.9 [28.7–72.3]	57.22 [23.7–72.3]	0.932
TRAP (20 μM)	69.6 [58.3–79.8]	83.3 [77.4–90.2]	**0.017**	68.3 [58.4–81]	75.7 [61–86.4]	0.509
EPI–ADP (0.1–0.25 μM)	22.4 [12.7–29.4]	26.8 [20.8–54.1]	0.092	16.6 [6.6–27.5]	24.2 [15.7–39.3]	0.359
CRP–XL (0.25 μg/mL)	79.6 [68.8–82.6]	86.9 [80.4–91.2]	**0.022**	71.8 [61.3–87.4]	82.9 [73.3–89.8]	0.141
***LAMP (CD63)***						
Unstimulated	6.9 [5.6–11]	6 [4.1–10]	0.514	6.9 [5.6–11]	8.8 [5.2–15.4]	0.406
ADP (0.5 μM)	31.2 [23.6–41.3]	25.8 [15.9–49.4]	0.446	28.3 [21.4–38.2]	31.5 [18.7–40.4]	0.717
ADP (20 μM)	62.2 [51.9–66.1]	57 [45.9–68.5]	0.786	58 [46.9–65]	57.9 [46.2–73.3]	0.594
TRAP (1.5 μM)	80.9 [7–85.9]	86.6 [74.2–91]	0.211	80.7 [68.3–87.8]	81.69 [67.7–88.9]	0.725
TRAP (20 μM)	93.6 [91.8–95.6]	96.1 [94.7–97.3]	**0.030**	94.2 [90.4–95.6]	95.8 [95.3–96.7]	**0.043**
EPI–ADP (0.1–0.25 μM)	42.7 [32.5–52]	44.4 [31.4–62]	0.415	43.8 [34.3–50]	37.00 [29.3–65.3]	0.865
CRP–XL (0.25 μg/mL)	95.1 [94.2–96.4]	95.5 [94.5–98]	0.384	94.8 [91.9–96.3]	96.6 [95.2–98]	**0.018**

Data are shown as median [IQR]. Levels of BDNF are measured by ng/mL. ADP, adenosine 5′ diphosphate; TRAP, the protease-activated receptor (PAR)-1 agonist thrombin receptor activator peptide; EPI, epinephrine; CRP-XL, the glycoprotein VI (GPVI) agonist collagen-related peptide.

**Table 3:** Comparison of patients with more than one bleeding symptom to those with one or no bleeding symptom.

	>1 Bleeding Symptom	One or No Bleeding Symptom	*p*-Value
**Number**	8	28	
**Mild genotype**	6	11	0.167
**Receiving GD-specific therapy**	7	26	0.99
**Platelet count (×10^3^/µL), mean ± SD**	183 ± 57.85	171 ± 97.56	0.66
**Thrombocytopenia (<50/<100/<150 × 10^3^/µL)**	0/2/2	0/1/9	
**Serum BDNF (ng/mL), mean ± SD**	7.66 ± 3.21	10.99 ± 4.0	**0.039**
**Plasma BDNF (ng/mL), median, IQR**	0.40 [0.29–0.64]	0.45 [0.33–0.77]	0.99

BDNF, brain-derived neurotrophic factor.

## Data Availability

Anonymized data may be available by request.

## References

[1] Revel-Vilk S., Szer J., Zimran A., Kaushansky K., Lichtman M., Prchal J., Levi M., Press O., Burns L., Caligiuri M. (2021). Gaucher disease and related lysosomal storage diseases. Williams Hematology.

[2] Patient Centered Guidelines for the Laboratory Diagnosis of Gaucher Disease (GD) Type 1. https://www.ewggd.com/wp-content/uploads/2022/09/Laboratory-diagnosis-of-Gaucher-disease-GD-type-1-.pdf.

[3] Serratrice C., Cherin P., Lidove O., Noel E., Masseau A., Leguy-Seguin V., Jaussaud R., Marie I., Lavigne C., Maillot F. (2019). Coagulation Parameters in Adult Patients With Type-1 Gaucher Disease. J. Hematol..

[4] Revel-Vilk S., Szer J., Zimran A. (2021). Hematological manifestations and complications of Gaucher disease. Expert Rev. Hematol..

[5] Simchen M.J., Oz R., Shenkman B., Zimran A., Elstein D., Kenet G. (2011). Impaired platelet function and peripartum bleeding in women with Gaucher disease. Thromb. Haemost..

[6] Spectre G., Roth B., Ronen G., Rosengarten D., Elstein D., Zimran A., Varon D., Revel-Vilk S. (2011). Platelet adhesion defect in type I Gaucher Disease is associated with a risk of mucosal bleeding. Br. J. Haematol..

[7] Giona F., Palumbo G., Amendola A., Santoro C., Mazzuconi M.G. (2006). Platelet function and coagulation abnormalities in type 1 Gaucher disease patients: Effects of enzyme replacement therapy (ERT). J. Thromb. Haemost..

[8] Ramstrom S., Sodergren A.L., Tynngard N., Lindahl T.L. (2016). Platelet Function Determined by Flow Cytometry: New Perspectives?. Semin. Thromb. Hemost..

[9] Boknas N., Macwan A.S., Sodergren A.L., Ramstrom S. (2019). Platelet function testing at low platelet counts: When can you trust your analysis?. Res. Pract. Thromb. Haemost..

[10] Revel-Vilk S., Naamad M., Frydman D., Freund M.R., Dinur T., Istaiti M., Becker-Cohen M., Falk R., Broide E., Michelson A.D. (2022). Platelet Activation and Reactivity in a Large Cohort of Patients with Gaucher Disease. Thromb. Haemost..

[11] Skaper S.D. (2012). The neurotrophin family of neurotrophic factors: An overview. Methods Mol. Biol..

[12] Fujimura H., Altar C.A., Chen R., Nakamura T., Nakahashi T., Kambayashi J., Sun B., Tandon N.N. (2002). Brain-derived neurotrophic factor is stored in human platelets and released by agonist stimulation. Thromb. Haemost..

[13] Azoulay D., Vachapova V., Shihman B., Miler A., Karni A. (2005). Lower brain-derived neurotrophic factor in serum of relapsing remitting MS: Reversal by glatiramer acetate. J. Neuroimmunol..

[14] Azoulay D., Urshansky N., Karni A. (2008). Low and dysregulated BDNF secretion from immune cells of MS patients is related to reduced neuroprotection. J. Neuroimmunol..

[15] Kerschensteiner M., Gallmeier E., Behrens L., Leal V.V., Misgeld T., Klinkert W.E., Kolbeck R., Hoppe E., Oropeza-Wekerle R.L., Bartke I. (1999). Activated human T cells, B cells, and monocytes produce brain-derived neurotrophic factor in vitro and in inflammatory brain lesions: A neuroprotective role of inflammation?. J. Exp. Med..

[16] Gejl A.K., Enevold C., Bugge A., Andersen M.S., Nielsen C.H., Andersen L.B. (2019). Associations between serum and plasma brain-derived neurotrophic factor and influence of storage time and centrifugation strategy. Sci. Rep..

[17] Nettiksimmons J., Simonsick E.M., Harris T., Satterfield S., Rosano C., Yaffe K., Health A.B.C.S. (2014). The associations between serum brain-derived neurotrophic factor, potential confounders, and cognitive decline: A longitudinal study. PLoS ONE.

[18] Azoulay D., Horowitz N.A. (2022). Brain-derived neurotrophic factor in hematological malignancies: From detrimental to potentially beneficial. Blood Rev..

[19] Serra-Millas M. (2016). Are the changes in the peripheral brain-derived neurotrophic factor levels due to platelet activation?. World J. Psychiatry.

[20] De Mello A.S., da Silva I.R., Reinaldo G.P., Dorneles G.P., Ce J., Lago P.D., Peres A., Elsner V.R., Coelho J.C. (2017). The modulation of inflammatory parameters, Brain-derived neurotrophic factor levels and global histone H4 acetylation status in peripheral blood of patients with Gaucher disease type 1. Clin. Biochem..

[21] Vairo F., Sperb-Ludwig F., Wilke M., Michellin-Tirelli K., Netto C., Neto E.C., Doederlein Schwartz I.V. (2015). Brain-derived neurotrophic factor expression increases after enzyme replacement therapy in Gaucher disease. J. Neuroimmunol..

[22] Amadio P., Porro B., Sandrini L., Fiorelli S., Bonomi A., Cavalca V., Brambilla M., Camera M., Veglia F., Tremoli E. (2019). Patho- physiological role of BDNF in fibrin clotting. Sci. Rep..

[23] Doyle E.L., Ridger V., Ferraro F., Turmaine M., Saftig P., Cutler D.F. (2011). CD63 is an essential cofactor to leukocyte recruitment by endothelial P-selectin. Blood.

[24] Im Y., Yoo H., Ko R.E., Lee J.Y., Park J., Jeon K. (2021). Exosomal CD63 in critically ill patients with sepsis. Sci. Rep..

[25] Israels S.J., McMillan-Ward E.M. (2005). CD63 modulates spreading and tyrosine phosphorylation of platelets on immobilized fibrinogen. Thromb. Haemost..

[26] Carmo L.A., Bonjour K., Ueki S., Neves J.S., Liu L., Spencer L.A., Dvorak A.M., Weller P.F., Melo R.C. (2016). CD63 is tightly associated with intracellular, secretory events chaperoning piecemeal degranulation and compound exocytosis in human eosinophils. J. Leukoc. Biol..

[27] Horowitz M., Braunstein H., Zimran A., Revel-Vilk S., Goker-Alpan O. (2022). Lysosomal functions and dysfunctions: Molecular and cellular mechanisms underlying Gaucher disease and its association with Parkinson disease. Adv. Drug Deliv. Rev..

[28] Chen A., Xiong L.J., Tong Y., Mao M. (2013). The neuroprotective roles of BDNF in hypoxic ischemic brain injury. Biomed. Rep..

[29] Almeida R.D., Manadas B.J., Melo C.V., Gomes J.R., Mendes C.S., Graos M.M., Carvalho R.F., Carvalho A.P., Duarte C.B. (2005). Neuroprotection by BDNF against glutamate-induced apoptotic cell death is mediated by ERK and PI3-kinase pathways. Cell Death Differ..

[30] Jiang L., Zhang H., Wang C., Ming F., Shi X., Yang M. (2019). Serum level of brain-derived neurotrophic factor in Parkinson’s disease: A meta-analysis. Prog. Neuropsychopharmacol. Biol. Psychiatry.

[31] Leiter O., Walker T.L. (2020). Platelets in Neurodegenerative Conditions-Friend or Foe?. Front. Immunol..

[32] Azoulay D., Naamad M., Frydman D., Broide E., Zimran A., Stemer G., Revel-Vilk S. (2022). P1633: Low serum BDNF levels are associated with lower platelet CD62P reactivity and increased bleeding tendency in patients with Gaucher disease. Hemasphere.

[33] Rodeghiero F., Tosetto A., Abshire T., Arnold D.M., Coller B., James P., Neunert C., Lillicrap D., ISTH/SSC joint VWF and Perinatal/Pediatric Hemostasis Subcommittees Working Group (2010). ISTH/SSC bleeding assessment tool: A standardized questionnaire and a proposal for a new bleeding score for inherited bleeding disorders. J. Thromb. Haemost..

[34] Rolfs A., Giese A.K., Grittner U., Mascher D., Elstein D., Zimran A., Bottcher T., Lukas J., Hubner R., Golnitz U. (2013). Glucosylsphingosine is a highly sensitive and specific biomarker for primary diagnostic and follow-up monitoring in Gaucher disease in a non-Jewish, Caucasian cohort of Gaucher disease patients. PLoS ONE.

